# Utility of serum chemokine–like factor 1 as a biomarker of severity and prognosis after severe traumatic brain injury: A prospective observational study

**DOI:** 10.1002/brb3.3522

**Published:** 2024-05-21

**Authors:** Xiaoyu Wu, Chang Su, Da Tian, Yufei Ye, Qinghua Du, Junxia Chen, Huguang Li, Jin Liu

**Affiliations:** ^1^ Department of Neurosurgery The Sixth Affiliated Hospital of Wenzhou Medical University Lishui China; ^2^ Department of Neurosurgery Lishui City People's Hospital Lishui China; ^3^ Department of Neurosurgery Qingyuan County People's Hospital Qingyuan China

**Keywords:** biomarkers, chemokine‐like factor 1, disease severity, mortality, prognosis, serum, severe traumatic brain injury

## Abstract

**Background:**

Chemokine‐like factor 1 (CKLF1) may be involved in the inflammatory response and secondary brain injury after severe traumatic brain injury (sTBI). We determined serum CKLF1 levels of sTBI patients to further investigate the correlation of CKLF1 levels with disease severity, functional prognosis, and 180‐day mortality of sTBI.

**Methods:**

Serum CKLF1 levels were measured at admission in 119 sTBI patients and at entry into study in 119 healthy controls. Serum CKLF levels of 50 patients were also quantified at days 1–3, 5, and 7 after admission. Glasgow coma scale (GCS) scores and Rotterdam computerized tomography (CT) classification were utilized to assess disease severity. Extended Glasgow outcome scale (GOSE) scores were recorded to evaluate function prognosis at 180 days after sTBI. Relations of serum CKLF1 levels to 180‐day poor prognosis (GOSE scores of 1–4) and 180‐day mortality were analyzed using univariate analysis, followed by multivariate analysis. Receiver‐operating characteristic (ROC) curve was built to investigate prognostic predictive capability.

**Results:**

Serum CKLF1 levels of sTBI patients increased at admission, peaked at day 2, and then gradually decreased; they were significantly higher during the 7 days after sTBI than in healthy controls. Differences of areas under ROC curve (areas under the curve [AUCs]) were not significant among the six time points. Multivariate analysis showed that serum CKLF1 levels were independently correlated with GCS scores, Rotterdam CT classification, and GOSE scores. Serum CKLF1 levels were significantly higher in non‐survivors than in survivors and in poor prognosis patients than in good prognosis patients. Serum CKLF1 levels independently predicted 180‐day poor prognosis and 180‐day mortality, and had high 180‐day prognosis and mortality predictive abilities, and their AUCs were similar to those of GCS scores and Rotterdam CT classification. Combination model containing serum CKLF1, GCS scores, and Rotterdam CT classification performed more efficiently than any of them alone in predicting mortality and poor prognosis. The models were visually described using nomograms, which were comparatively stable under calibration curve and were relatively of clinical benefit under decision curve.

**Conclusion:**

Serum CKLF1 levels are significantly associated with disease severity, poor 180‐day prognosis, and 180‐day mortality in sTBI patients. Hence, complement CKLF1 may serve as a potential prognostic biomarker of sTBI.

## 1 INTRODUCTION

Traumatic brain injury (TBI) is a common neurosurgical condition (Capizzi et al., 2020). Patients with post‐traumatic Glasgow coma scale (GCS) scores ≤8 are considered to suffer from severe TBI (sTBI) (Mostert et al., 2022). The GCS and the Rotterdam computerized tomography (CT) classification are the two clinically valuable prognostic factors of sTBI (Abeytunge et al., 2021). Secondary brain injury is one of the important causes affecting the prognosis of sTBI, and its main pathological mechanisms include inflammation, cellular death, cytotoxic effects, and oxidative stress (Liu et al., 2015; Ni et al., 2022). Prognosis prediction is a pivotal step during the treatment of sTBI, and circulating biomarkers have also been studied as the potential predictors of clinical outcomes after sTBI during recent decades.

Chemokine‐like factor 1 (CKLF1) is a member of the chemokine‐like family, which has the structural characteristics and functions of chemokines (Cai et al., 2020). CKLF1 is in possession of the potential abilities that are similar to those of chemotactic monocytes, neutrophils, and lymphocytes (Han et al., 2001). Current studies have indicated that CKLF1 may play an important role in inflammatory diseases, such as atherosclerosis, rheumatoid arthritis, and pneumonia (Tao et al., 2016; Tian et al., 2011; Zhang et al., 2013). Excitingly, CKLF1 was significantly expressed in rat ischemic brain tissues and involved in the process of secondary brain injury after ischemic stroke (Chen, Chu et al., 2019; Zhou et al., 2022). Interestingly, inhibition of CKLF1 could markedly reduce inflammatory response after acute brain injury, thereby lessening the destruction of blood–brain barrier and subsequently protecting neurologic function (Ai, Chen, Chu, Luo et al., 2019; Kong et al., 2016). Therefore, it is speculated that CKLF1 may represent a potential biomarker of acute brain injury. In this study, we sought to determine whether serum CKLF1 levels are associated with the severity and prognosis of patients with sTBI.

## 2 MATERIALS AND METHODS

### 2.1 Study design, inclusion and exclusion criteria, and participant enrollments

In this prospective observational study, isolated and blunt sTBI patients who were admitted to Lishui City People's Hospital (Lishui, China) within 12 h after the injury from May 2020 to June 2022, were consecutively enrolled. We excluded those patients with (1) age <18 years; (2) incomplete information; (3) loss to follow‐up; (4) refusal to participate; (5) unavailable blood samples; (6) infection or operational procedures in the last month; (7) previous neurological diseases (such as ischemic stroke, intracerebral hemorrhage, and TBI); (8) pregnancies or other severe diseases (e.g., malignant tumors, uremia, cirrhosis, and liver or heart failure). The healthy control group was consecutively recruited in the physical examination center of Lishui People's Hospital during the same period. This study is completed based on the principles of the Declaration of Helsinki, and its protocol was approved by the Ethics Committee at the Lishui City People's Hospital (Opinion No.: Medical Ethics Review No. 2020‐001). Written informed consent to participate was obtained from controls themselves or patients’ relatives.

### 2.2 Study variable investigation, blood sample collection and immune analysis

We collected some relevant information, including age, gender, previous underlying diseases (such as hypertension, diabetes mellitus, and hyperlipidemia), cigarette smoking, alcohol consumption, indicators of trauma severity, radiological parameters, admission time, blood collection time, vital signs, and trauma mechanisms. Clinical severity was evaluated using GCS scores, and radiological severity was assessed via Rotterdam CT classification. Radiological parameters included midline shift, abnormal cistern, epidural hematoma, subdural hematoma, subarachnoid hemorrhage, intraventricular hemorrhage, intracerebral hemorrhage, brain contusion, and pneumocranium. Patients were followed until death or the completion of 180 days after injury, and functional prognosis was assessed using the extended Glasgow outcome scale (GOSE). A poor prognosis was defined as GOSE scores of 1–4 (Yan et al., 2022).

Blood samples were collected from patients at admission, and those of controls were obtained at entry into the study. In addition, a portion of patients consented for blood collections at days 1–3, 5, and 7 after admission. Laboratory indices, such as blood leukocyte count, were measured using the conventional methods. For the sake of the determination of CKLF1 levels, acquired serum samples were stored at −80°C until assayed. Serum CKLF1 levels were quantified by a commercially available enzyme‐linked immunosorbent assay (Shuhua Biotechnology) according to the manufactures’ instructions. Its detection range was from 1.0 to 480 pg/mL, the detection sensitivity was 1.0 pg/mL, and intra‐assay coefficients of variation were <15% and inter‐assay coefficients of variation were <15%. All measurements were performed in duplicate by the same technician who was blinded to the clinical data, and two measurements were averaged for final analysis.

### 2.3 Statistical analysis

All statistical analyses were performed using Statistical Package for the Social Sciences version 26.0 (SPSS Inc.) and MedCalc version 9.6.4.0 (MedCalc Software). Graphs were drawn using GraphPad Prism (version 9.0; GraphPad Software Inc.) and R software (version 4.2.2; https://www.r‐project.org). Qualitative variables are expressed as frequency (percentage). The Kolmogorov–Smirnov test was performed to determine the normal distribution of quantitative data. Quantitative data of normal distribution are expressed as mean ± standard deviation, and quantitative data of non‐normal distribution are expressed in the form of median (upper and lower quartiles). The Mann–Whitney *U* test or *t* test was used for comparison of quantitative data, and the *χ* ^2^ test or Fisher's exact test, for qualitative data comparison. The Spearman correlation coefficient was performed to assess bivariate correlations. Additionally, the multivariate linear regression model was established to identify the variables, which were independently correlated with serum CKLF1 levels and GOSE scores. The binary logistic regression model was built to investigate whether serum CKLF1 levels were associated with poor prognosis at 180 days after trauma. The associations were expressed as odds ratios and 95% confidence intervals (CIs). A Cox proportional risk model was constructed to determine the relationship between serum CKLF1 levels and 180‐day overall survival. Results are expressed as hazard ratio and 95% CI. Subsequently, receiver‐operating characteristic (ROC) curves were plotted to investigate the predictive value of serum CKLF1 levels for 180‐day poor prognosis and mortality in sTBI patients and estimate the areas under the curve (AUCs). The Youden‐J method was used to determine cutoff values and generate the corresponding sensitivity and specificity values. Combination models were graphically represented by nomograms, and calibration curves were drawn to verify the stability of the model. Two‐sided *p* < .05 indicated statistical significance.

## 3 RESULTS

### 3.1 Clinical features of sTBI patients and controls

A total of 153 sTBI patients were initially enrolled in this study. Subsequently, 34 patients were excluded for the reasons outlined in Figure S1. Finally, a total of 119 patients were enrolled in the current study. There were 65 males and 54 females, who were aged from 18 to 77 years (median, 46 years; upper–lower quartiles, 33–57 years). Of the 34 excluded patients, there were 19 males and 15 females aged from 22 to 78 years (median, 50 years; upper–lower quartiles, 42–57 years). There were no significant differences in age and gender ratio between the included patients and the excluded ones (both *p* > .05). In addition, 119 healthy volunteers were recruited as controls, including 61 males and 58 females, who were aged from 22 to 79 years (median 49 years; upper–lower quartiles, 36–59 years). The age and sex ratio of control group were similar to those of patient group (both *p* > .05).

Among this cohort of patients, 27 (22.7%) were cigarette smokers, 34 (28.6%) were alcohol drinkers, 21 (17.6%) suffered from hypertension, 17 (14.3%) had diabetes, and 25 (21.0%) were inflicted with hyperlipidemia. Hospital admission time ranged from 0.5 to 12.0 h after injury (median, 4.2 h; upper–lower quartiles, 3.1–5.6 h), and the range of blood collection time was 0.9–13.3 h (median, 5.7 h; upper–lower quartiles, 4.1–7.5 h). Systolic arterial pressures ranged from 71 to 184 mmHg (median, 122 mmHg; upper–lower quartiles, 99–137 mmHg), and diastolic arterial pressures ranged from 43 to 106 mmHg (median, 74 mmHg; upper–lower quartiles, 65–84 mmHg). Causes of trauma included car/motorcycle in 61 (51.3%), fall/jump in 46 (38.7%), and others in 12 (10.1%). Patients’ GCS scores ranged from 3 to 8 (median, 5; upper–lower quartiles, 4–7), with scores 3–8 in 15 (12.6%), 25 (21.0%), 31 (26.1%), 18 (15.1%), 14 (11.8%), and 16 (13.4%) patients, respectively; and Rotterdam CT classification ranged from 3 to 6 (median, 4; upper–lower quartiles, 4–5), with score 3 in 17 (14.3%) patients, score 4 in 53 (44.5%), score 5 in 29 (24.4%), and score 6 in 20 (16.8%). There were 66 (55.5%) patients with midline shift >5 mm, 90 (75.6%) with abnormal cisterns, 64 (53.8%) with epidural hematomas, 69 (58.0%) with subdural hematomas, 77 (64.7%) with subarachnoid hemorrhages, 13 (10.9%) with intraventricular hemorrhages, 67 (56.3%) with intracerebral hemorrhages, 71 (59.7%) with brain contusions, and 46 with pneumocephalus (38.7%). Blood leukocyte counts ranged from 3.6 to 18.1 × 10^9^/L (median, 9.3 × 10^9^/L; upper–lower quartiles, 6.8–11.0 × 10^9^/L).

### 3.2 Serum CKLF1 levels and severity of sTBI

**FIGURE 1:**
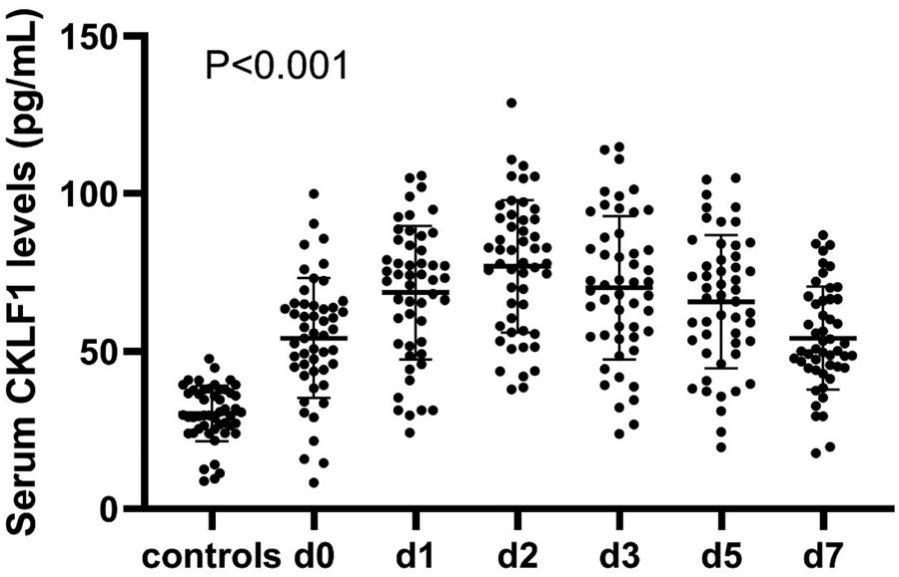
Dynamic change of serum chemokine‐like factor 1 (CKLF1) levels after severe traumatic brain injury. Serum CKLF1 levels of patients increased at day 0 (at admission), peaked at day 2, and then gradually decreased and were significantly higher during 7 days than those of healthy controls (*p* < .001).

**FIGURE 2:**
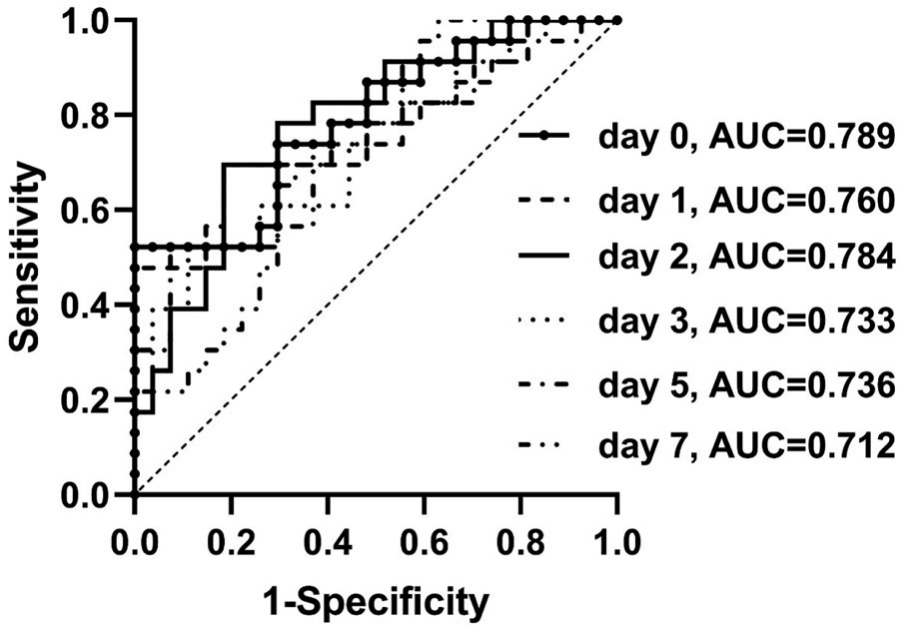
Areas under receiver‐operating characteristic (ROC) curve of serum chemokine‐like factor 1 levels at admission and at days 1–3, 5, and 7 for predicting 180‐day poor prognosis after severe traumatic brain injury. Areas under ROC curve were.786,.760,.784,.733,.716, and.732 at admission and at days 1–3, 5, and 7 after severe brain injury, respectively, and differences of areas under the curve (AUCs) were not significant among the six time points (all *p* > .05).

Serum CKLF levels were measured at days 0–3, 5, and 7 after admission in 50 patients of a total of 119 sTBI patients. Serum CKLF1 levels of sTBI patients increased at admission, peaked at day 2, and then gradually decreased and were significantly higher during the 7 days after sTBI than in healthy controls (*p* < .001; Figure 1). AUCs of serum CKLF1 levels at admission and days 1–3, 5, and 7 were.786 (95% CI,.650–.892),.760 (95% CI,.618–.869),.784 (95% CI,.645–.888),.733 (95% CI,.589–.848),.716 (95% CI,.592–.850), and.732 (95% CI,.566–.831), respectively, and differences of AUCs were not significant among the six time points (all *p* > .05; Figure 2). In Figure S2, among all 119 patients and 119 controls, serum CKLF1 levels were significantly higher in patients (range, 8.35–99.92 pg/mL; median, 55.08 pg/mL; upper–lower quartiles, 44.03–64.43 pg/mL) than in controls (range, 8.76–47.71 pg/mL; median, 31.68 pg/mL, upper–lower quartiles, 26.38–36.85 pg/mL).

For the sake of investigating the relationship between serum CKLF1 levels and trauma severity indicated by GCS score and Rotterdam CT classification, GCS scores and Rotterdam CT classification were in advance defined as the categorical and continuous variables. In Table 1, Spearman's correlation analysis showed a significantly negative correlation between serum CKLF1 levels and GCS scores (*p* < .001), as well as a significantly positive correlation with Rotterdam CT classification (*p* < .001). Meanwhile, serum CKLF1 levels were significantly correlated with midline shift >5 mm, abnormal cisterns, epidural hemorrhage, and blood leukocyte count (all *p* < .05). In Table 2, GCS scores and Rotterdam CT classification were independently correlated with serum CKLF1 levels (both *p* < .05). Subsequently, Figure S3 shows the close relations of serum CKLF1 levels with both GCS scores and Rotterdam CT classification, which were regarded as whether the continuous or categorical variables (all *p* < .001).

**TABLE 1:** Factors correlated with serum chemokine‐like factor 1 levels after severe traumatic brain injury.

	*ρ*	*p* Value
Gender (male/female)	−.037	.693
Age (year)	.104	.261
Current cigarette smoking	.173	.060
Alcohol abuse	.089	.334
Hypertension	.065	.479
Diabetes mellitus	.080	.389
Hyperlipidemia	.062	.500
Hospital admission time (h)	−.133	.149
Blood‐sampling time (h)	−.140	.130
Systolic arterial pressure (mmHg)	−.174	.058
Diastolic arterial pressure (mmHg)	−.102	.270
Traumatic causes	−.108	.242
GCS scores	−.589	*<.001
Rotterdam CT classification	.578	*<.001
Midline shift >5 mm	.228	*.013
Abnormal cisterns	.225	*.014
Epidural hematoma	.310	*.001
Subdural hematoma	.170	.064
Subarachnoid hemorrhage	.151	.102
Intraventricular hemorrhage	.029	.757
Intracerebral hematoma	.090	.333
Brain contusion	.101	.276
Pneumocephalus	.038	.678
Blood leucocyte count (×10^9^/L)	.236	*.010

*Note*: Correlations were analyzed using the Spearman test.

Abbreviations: CT, computerized tomography; GCS, Glasgow coma scale.

P < 0.05.

**TABLE 2:** Correlation between serum chemokine‐like factor 1 levels and other variables using linear regression analysis in severe traumatic brain injury.

	Univariate linear regression		Multivariate linear regression	
	*t*	*p* Value	*t*	*p* Value
Gender (male/female)	−.870	.386	–	–
Age (year)	1.183	.239	–	–
Current cigarette smoking	1.498	.137	–	–
Alcohol abuse	.901	.369	–	–
Hypertension	.333	.740	–	–
Diabetes mellitus	.821	.414	–	–
Hyperlipidemia	.840	.402	–	–
Hospital admission time (h)	−1.566	.120	–	–
Blood‐sampling time (h)	−1.723	.088	–	–
Systolic arterial pressure (mmHg)	−1.801	.074	–	–
Diastolic arterial pressure (mmHg)	−.733	.465	–	–
Traumatic causes	−.417	.678	–	–
GCS scores	−7.695	*<.001	−2.574	*.011
Rotterdam CT classification	8.054	*<.001	3.178	*.002
Midline shift >5 mm	2.151	*.034	.202	.840
Abnormal cisterns	2.395	*.018	.895	.373
Epidural hematoma	3.236	*.002	1.575	.118
Subdural hematoma	1.220	.225	–	–
Subarachnoid hemorrhage	1.234	.220	–	–
Intraventricular hemorrhage	.436	.664	–	–
Intracerebral hematoma	.816	.416	–	–
Brain contusion	.850	.397	–	–
Pneumocephalus	.600	.550	–	–
Blood leucocyte count (×10^9^/l)	2.583	*.011	1.088	.279

*Note*: Correlations were done using a linear regression model in severe traumatic brain injury.

Abbreviations: CT, computerized tomography; GCS, Glasgow coma scale.

P < 0.05.

### 3.3 Relationship between serum CKLF1 levels and GOSE scores after sTBI

As shown in Table S1, GOSE scores were significantly correlated with serum CKLF1 levels, GCS scores and Rotterdam CT classification, midline shift >5 mm, abnormal cisterns, epidural hemorrhage, subdural hemorrhage, and blood leukocyte counts (all *p* < .05). In Table S2, GCS scores, Rotterdam CT classification, and serum CKLF1 levels were independently correlated with 180‐day GOSE scores (all *p* < .05). Subsequently, serum CKLF1 levels had intimate correlations with GOSE scores, regardless of whether GOSE was a continuous or categorical variable (both *p* < .001; Figure S4).

### 3.4 Serum CKLF1 levels and 180‐day death after sTBI

**FIGURE 3:**
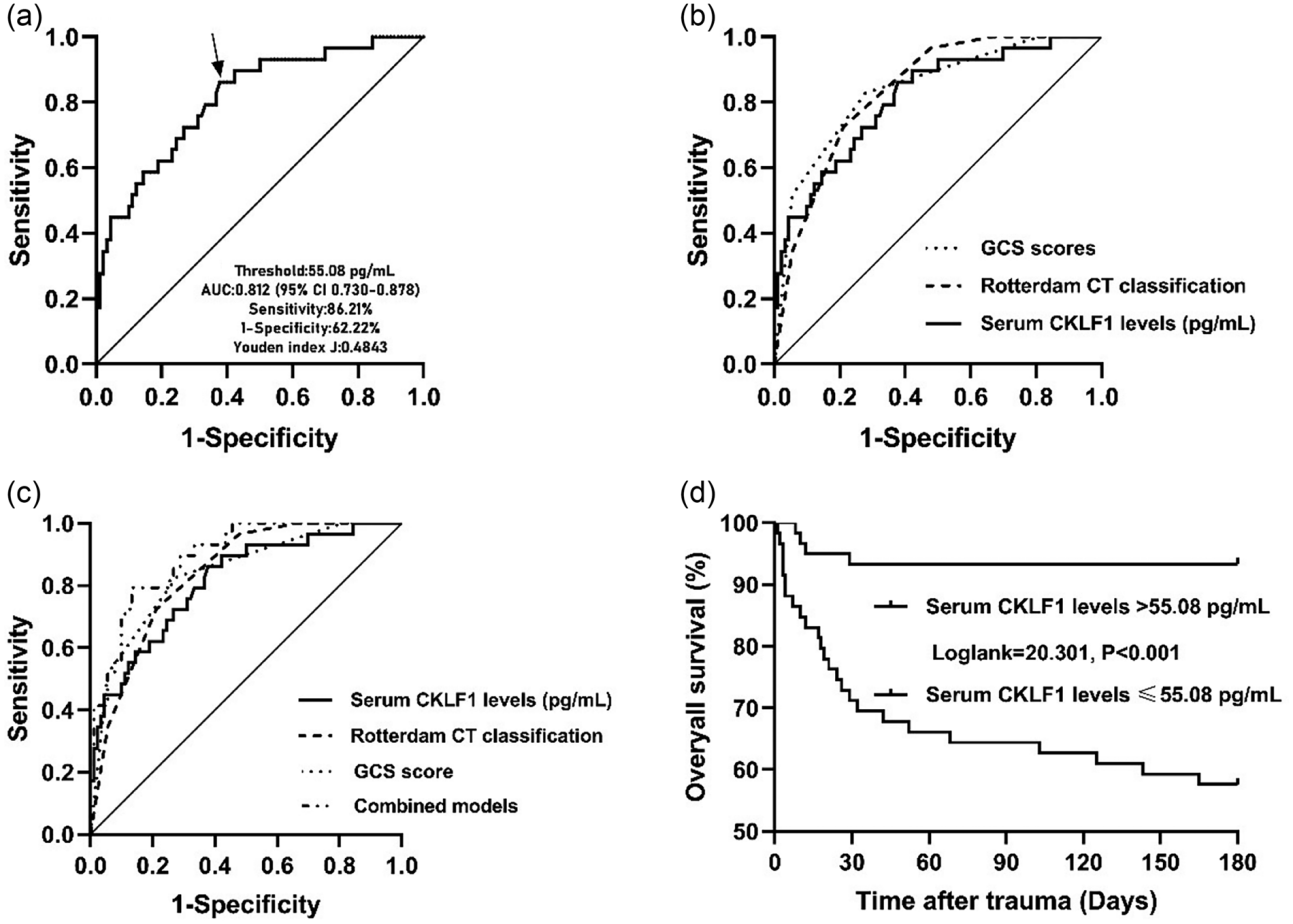
Receiver‐operating characteristic curve and overall survival curve showing relationship between admission serum chemokine‐like factor 1 (CKLF1) levels and 180‐day death after severe traumatic brain injury. (a) Receiver‐operating characteristic curve for admission serum CKLF1 levels used to predict 180‐day mortality after severe traumatic brain injury. Admission serum CKLF1 levels significantly predicted 180‐day mortality after severe traumatic brain injury (*p* < .001). Its optimal level was identified, which predicted mortality with the maximum Youden index. (b) Receiver‐operating characteristic curve for admission serum CKLF1 levels, baseline Glasgow coma scale (GCS) scores, and baseline Rotterdam computerized tomography (CT) classification used to predict 180‐day mortality after severe traumatic brain injury. The post‐traumatic 180‐day death predictive ability of admission serum CKLF1 levels was similar to those of baseline GCS scores, and baseline Rotterdam CT classification (both *p* > .05). (c) Receiver‐operating characteristic curve for admission serum CKLF1 levels, baseline GCS scores, baseline Rotterdam CT classification, and the combined model used to predict 180‐day mortality after severe traumatic brain injury. The combined model was composed of admission serum CKLF1 levels, baseline GCS scores and baseline Rotterdam CT classification. The post‐traumatic 180‐day death predictive ability of the combined model was significantly stronger than those of admission serum CKLF1 levels, baseline GCS scores, and baseline Rotterdam CT classification alone (all *p* < .05). (d) A 180‐day overall survival curve for comparing survival time and probability between subgroups with admission serum CKLF1 levels above or below the cutoff value under receiver‐operating characteristic curve among severe traumatic brain injury patients. Overall survival time was significantly shorter in patients with admission serum CKLF1 levels >55.08 pg/mL than in the other remainders (*p* < .001). AUC, area under the curve.

The mean 180‐day overall survival time of patients was 144.5 days (95% CI, 132.6–156.4 days). A total of 29 patients died within 180 days of the onset of head trauma. As shown in Figure S5, serum CKLF1 levels were significantly higher in the deceased than in the alive (*p* < .001). Other variables, that is, GCS scores, Rotterdam CT classification, midline shift >5 mm, abnormal cisterns, subdural hematoma, blood leukocyte count, and serum CKLF1 levels, were significantly different between the two groups (all *p* < .05; Table S3). In Table 3, GCS scores, Rotterdam CT classification, and serum CKLF1 levels were independently associated with patients’ 180‐day overall survival (all *p* < .05). Subsequently, ROC curve analysis showed that serum CKLF1 levels significantly predicted the occurrence of 180‐day death (AUC,.812; 95% CI,.730–.878; Figure 3a). Serum CKLF1 levels distinguished patients at risk of 180‐day death with 86.21% sensitivity and 62.22% specificity (maximum Youden index J,.4843). Interestingly, its predictive ability was similar to those of GCS scores (AUC = .837; 95% CI,.758–.898; *p* = .636) and Rotterdam CT classification (AUC = .840; 95% CI,.762–.901; *p* = .613) (Figure 3b). Subsequently, we established a prediction model comprising serum CKLF1, GCS scores, and Rotterdam CT classification, and it was found that the predictive ability of the combined model (AUC = .893; 95% CI,.823–.942) was significantly higher than those of serum CKLF1 levels (*p* = .045), GCS scores (*p* = .024), and Rotterdam CT classification (*p* = .024; Figure 3c). Using the cutoff value of 55.08 pg/mL under ROC curve, all patients were categorized into two groups, respectively, with serum CKLF1 levels >55.08 pg/mL and ≤55.08 pg/mL. As shown in Figure 3d, the survival time of patients with serum CKLF1 levels >55.08 pg/mL was significantly lower, as compared to patients with lower levels of CKLF1 (*p* < .01). As shown in Figure S6, the significantly correlated variables in the multivariate logistic regression analysis were forced into the nomogram model to predict the associated risks. The points corresponding to the above three variables were summed to calculate the total points, and different points corresponded to different survival probabilities, which were 0.9 = 113, 0.7 = 145, 0.5 = 162, 0.3 = 176, and 0.1 = 192. In addition, the configured calibration curve confirmed that there is medium–high stability for such a model (Figure S7).

**TABLE 3:** Univariate and multivariate Cox's proportional hazard regression analysis of predictors for 180‐day overall survival after severe traumatic brain injury.

	Univariate analysis		Multivariate analysis	
	Hazard ratio (95% *CI*)	*p* Value	Hazard ratio (95% *CI*)	*p* Value
Gender (male/female)	1.021 (.491–2.122)	.956	–	–
Age (year)	1.012 (.987–1.037)	.358	–	–
Current cigarette smoking	1.310 (.580–2.959)	.515	–	–
Alcohol abuse	1.412 (.667–2.990)	.854	–	–
Hypertension	1.917 (.849–4.330)	.117	–	–
Diabetes mellitus	1.645 (.670–4.040)	.278	–	–
Hyperlipidemia	.764 (.292–2.004)	.585	–	–
Hospital admission time (h)	.915 (.789–1.062)	.244	–	–
Blood‐sampling time (h)	.905 (.787–1.039)	.157	–	–
Systolic arterial pressure (mmHg)	.995 (.982–1.008)	.441	–	–
Diastolic arterial pressure (mmHg)	.997 (.974–1.021)	.823	–	–
Traumatic causes	.948 (.541–1.664)	.853	–	–
GCS scores	.392 (.273–.563)	*<.001	.578 (.376–.890)	*.013
Rotterdam CT classification	3.929 (2.466–6.260)	*<.001	2.243 (1.257–4.004)	*.006
Midline shift >5 mm	2.334 (1.033–5.272)	*.041	1.060 (.447–2.514)	.895
Abnormal cisterns	4.802 (1.167–20.648)	*.032	2.379 (.533–10.618)	.256
Epidural hematoma	2.454 (1.086–5.541)	*.031	.730 (.305–1.745)	.479
Subdural hematoma	1.654 (.753–3.632)	.210	–	–
Subarachnoid hemorrhage	1.820 (.777–4.261)	.168	–	–
Intraventricular hemorrhage	1.848 (.705–4.844)	.212	–	–
Intracerebral hematoma	1.824 (.831–4.077)	.134	–	–
Brain contusion	1.143 (.540–2.420)	.727	–	–
Pneumocephalus	1.192 (.569–2.495)	.642	–	–
Blood leucocyte count (×10^9^/L)	1.128 (1.011–1.258)	*.032	1.145 (.999–1.313)	.051
Serum CKLF1 levels (pg/mL)	1.076 (1.049–1.104)	*<.001	1.037 (1.002–1.074)	*.037

*Note*: Results were presented as odds ratios (95% confidence interval) using the univariate and multivariate Cox's proportional hazard regression analyses.

Abbreviations: CI, confidence interval; CT, computerized tomography; CKLF1, chemokine‐like factor 1; GCS, Glasgow coma scale.

P < 0.05.

### 3.5 Serum CKLF1 levels and poor 180‐day prognosis after sTBI

**FIGURE 4:**
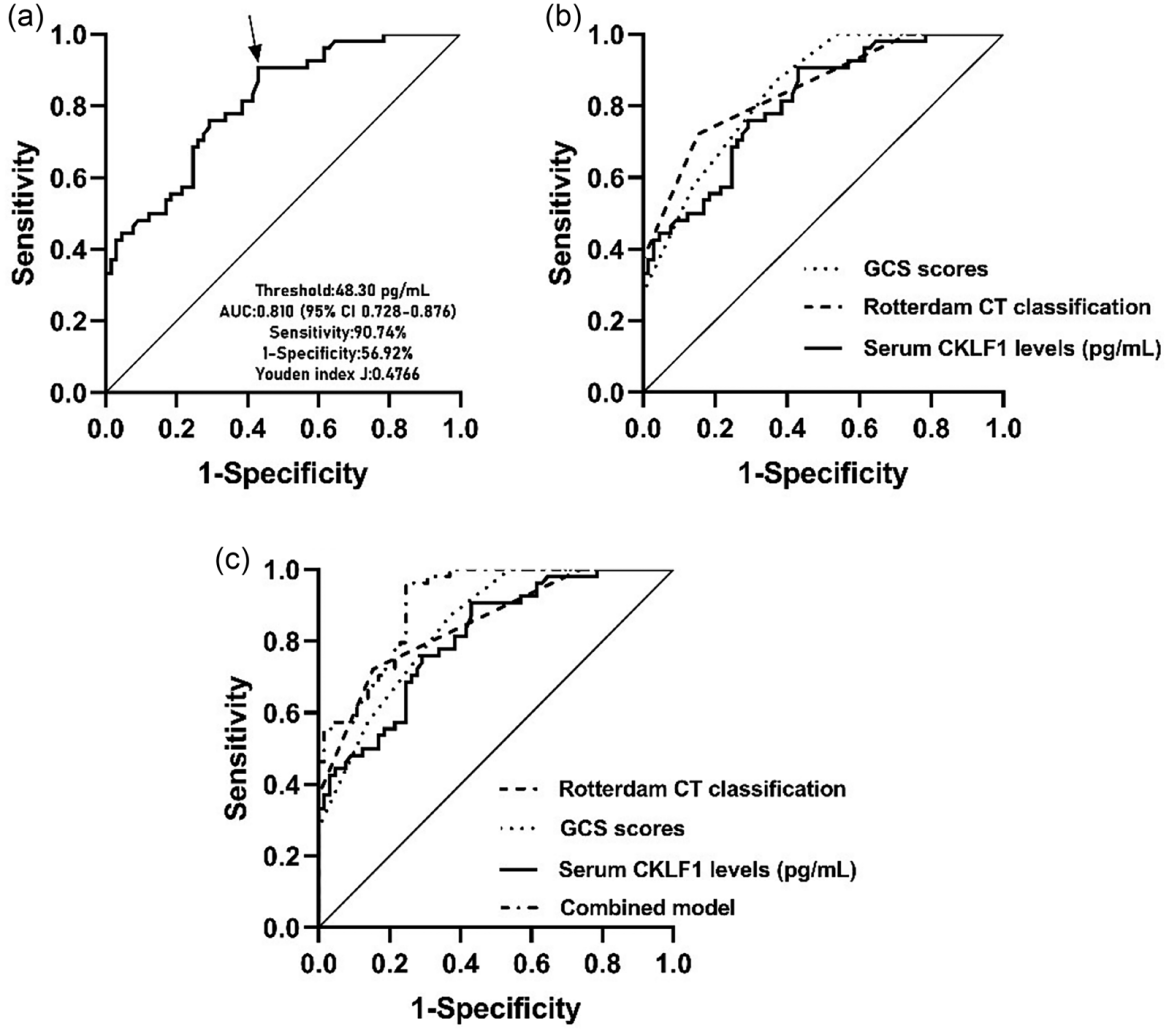
Receiver‐operating characteristic curve of admission serum chemokine‐like factor 1 (CKLF1) levels with respect to 180‐day poor prognosis predictive ability among patients with severe traumatic brain injury. (a) Receiver‐operating characteristic curve for admission serum CKLF1 levels used to predict 180‐day poor prognosis after severe traumatic brain injury. Admission serum CKLF1 levels significantly predicted 180‐day poor prognosis after severe traumatic brain injury (*p* < .001). Its optimal level was identified, which predicted 180‐day poor prognosis with the maximum Youden index. (b) Receiver‐operating characteristic curve for admission serum CKLF1 levels, baseline Glasgow coma scale (GCS) scores, and baseline Rotterdam computerized tomography (CT) classification used to predict 180‐day poor prognosis after severe traumatic brain injury. The post‐traumatic 180‐day poor prognosis predictive ability of admission serum CKLF1 levels was similar to those of baseline GCS scores and baseline Rotterdam CT classification (both *p* > .05). (c) Receiver‐operating characteristic curve for admission serum CKLF1 levels, baseline GCS scores, baseline Rotterdam CT classification, and the combined model used to predict 180‐day poor prognosis after severe traumatic brain injury. The combined model was composed of admission serum CKLF1 levels, baseline GCS scores, and baseline Rotterdam CT classification. The post‐traumatic 180‐day poor prognosis predictive ability of the combined model was significantly stronger than those of admission serum CKLF1 levels, baseline GCS scores, and baseline Rotterdam CT classification alone (all *p* < .05). AUC, area under the curve.

A total of 45 patients had a poor prognosis (GOSE scores 1–4) at 180 days after sTBI. As shown in Figure S8, serum CKLF1 levels were significantly higher in the patients who suffered poor prognosis than those who had good prognosis (*p* < .001). In Table S4, GCS scores, Rotterdam CT classification, midline shift >5 mm, abnormal cisterns, subdural hematoma, blood leukocyte count, and serum CKLF1 levels differed significantly between the two groups (all *p* < .05). Subsequently, in Table 4, GCS scores, Rotterdam CT classification, and serum CKLF1 levels were independently associated with poor prognosis at 180 days after sTBI (all *p* < .05). Under ROC curve, serum CKLF1 levels significantly predicted the occurrence of 180‐day poor prognosis (AUC,.810; 95% CI,.728–.876; Figure 3a). Serum CKLF1 levels distinguished patients at risk of 180‐day poor prognosis with 90.74% sensitivity and 56.92% specificity (maximum Youden index J,.4766; Figure 4a), and interestingly, its predictive ability was similar to the GCS scores (AUC = .845; 95% CI,.768–.905; *p* = .388) and Rotterdam CT classification (AUC = .849; 95% CI,.772–.908; *p* = .372) (Figure 4b). Subsequently, we built a combined prediction model with serum CKLF1 levels, GCS scores, and Rotterdam CT classification and found that the predictive ability of the combined model (AUC = .907; 95% CI,.840–.953) was significantly higher than those of serum CKLF1 levels (*p* = .002), GCS scores (*p* = .005), and Rotterdam CT classification (*p* = .009) (Figure 4c). As shown in Figure S9, we included significantly correlated variables from multivariate logistic regression analysis into the nomogram model to predict the associated risks. In addition, the points corresponding to the above three variables were summed to calculate the total points, and different points corresponded to different risks, which were 0.1 = 93.2, 0.3 = 119.5, 0.5 = 136.0, 0.7 = 152.5, and 0.9 = 178.8, respectively. In addition, the configured calibration curve confirmed that there was medium–high stability for such a model (Figure S10).

**TABLE 4:** Univariate and multivariate logistic regression analysis of predictors of 180‐day poor prognosis after severe traumatic brain injury.

	Univariate analysis		Multivariate analysis	
	Odds ratio (95% *CI*)	*p* Value	Odds ratio (95% *CI*)	*p* Value
Gender (male/female)	.934 (.453–1.929)	.855	–	–
Age (year)	1.006 (.982–1.031)	.624	–	–
Current cigarette smoking	1.400 (.592–3.309)	.443	–	–
Alcohol abuse	1.211 (.556–2.636)	.861	–	–
Hypertension	1.778 (.686–4.608)	.236	–	–
Diabetes mellitus	1.425 (.509–3.989)	.500	–	–
Hyperlipidemia	.932 (.384–2.264)	.876	–	–
Hospital admission time (h)	.923 (.804–1.060)	.257	–	–
Blood‐sampling time (h)	.922 (.808–1.051)	.224	–	–
Systolic arterial pressure (mmHg)	.990 (.977–1.003)	.145	–	–
Diastolic arterial pressure (mmHg)	.986 (.963–1.010)	.243	–	–
Traumatic causes	1.383 (.802–2.386)	.244	–	–
GCS scores	.300 (.194–.465)	*<.001	.511 (.278–.941)	*.031
Rotterdam CT classification	8.509 (4.012–18.050)	*<.001	4.069 (1.571–10.536)	*.004
Midline shift >5 mm	2.702 (1.271–5.744)	*.010	1.286 (.405–4.082)	.669
Abnormal cisterns	4.381 (1.629–11.782)	*.003	4.201 (.959–18.403)	.057
Epidural hematoma	2.643 (1.250–5.590)	*.011	1.121 (.354–3.552)	.846
Subdural hematoma	1.939 (.920–4.089)	.082	–	–
Subarachnoid hemorrhage	2.162 (.989–4.726)	.053	–	–
Intraventricular hemorrhage	1.465 (.461–4.652)	.518	–	–
Intracerebral hematoma	1.648 (.790–3.441)	.183	–	–
Brain contusion	1.984 (.935–4.213)	.074	–	–
Pneumocephalus	.764 (.363–1.610)	.479	–	–
Blood leucocyte count (×10^9^/L)	1.155 (1.020–1.307)	*.023	1.069 (.886–1.290)	.488
Serum CKLF1 levels (pg/mL)	1.099 (1.058–1.142)	*<.001	1.050 (1.001–1.101)	*.044

*Note*: Results were presented as odds ratios (95% confidence interval) using the univariate and multivariate logistic regression analyses.

Abbreviations: CI, confidence interval; CT, computerized tomography; CKLF1, chemokine‐like factor 1; GCS, Glasgow coma scale.

P < 0.05.

## 4 DISCUSSION

sTBI is a life‐threatening disease. Diagnosis, treatment, and prognostic prediction are important components of clinical work in sTBI. GCS scores and Rotterdam CT classification are often used to assess the severity of patients and are common prognostic predictors, which have been validated in many previous studies (Ketharanathan et al., 2023). In this study, we found that serum CKLF1 levels were significantly higher in sTBI patients compared with controls; interestingly, we also found that (1) serum CKLF1 levels in sTBI patients were significantly negatively correlated with patients’ GCS scores and were positively correlated with Rotterdam CT classification; (2) serum CKLF1 levels in sTBI patients were significantly negatively correlated with 180‐day GOSE scores, and serum CKLF1 was an independent predictor of death and poor prognosis at 180 days after sTBI; (3) serum CKLF1 can effectively predict the occurrence of poor prognosis in sTBI patients. In conclusion, elevated serum CKLF1 levels are closely related to the severity of the disease and the clinical prognosis of sTBI patients. It is suggested that serum CKLF1 may be a potential prognosis biomarker for sTBI.

Inflammatory response plays an important role in acute brain injury diseases and participates in related pathophysiological processes (Corps et al., 2015). CKLF1 has the potential to function as chemotaxis monocytes, neutrophils, and lymphocytes; and overproduction of CKLF1 activates the inflammatory response and exacerbates the impairment of neurological function (Han et al., 2001). One study found that CKLF1 expression was significantly elevated in the cortex and hippocampus of transient middle cerebral artery occlusion rats (Kong et al., 2011). When the specific inhibitor C19 was used to restrain CKLF1, infarct foci were reduced and neurological function was improved in experimental animals compared to the controls (Kong et al., 2012, 2011), which may be achieved by reducing the infiltration of neutrophils around the infarct foci (Kong et al., 2014). Further in vitro experiments revealed that the CKLF1/CC chemokine receptor (CCR)4 as well as CKLF1/CCR5 axes are involved in the migration process of neutrophils (Chen et al., 2020). When specifically inhibiting the CKLF1/CCR4 axis, the activation of NLRP3 and the subsequent inflammatory response process could be further inhibited, thus protecting neurological function and improving cerebral ischemia (Ai et al., 2019). Meanwhile, the effect of CKLF1 on inflammatory response was also reflected in the regulation of microglia polarization. Specifically, when brain tissue suffered from ischemia/reperfusion injury, the expression of CKLF1 in BV2 microglia was increased, and increased CKLF1 induced the polarization of BV2 microglia to the M1 phenotype and induced the process of inflammatory response (Chen et al., 2019). Reportedly, CKLF1 role in ischemic stroke may be reflected in increased blood–brain barrier disruption (Kong et al., 2016; Wu et al., 2023). After injecting CKLF1 antibody into the right encephalocele of cerebral ischemic rats, brain water content was decreased, expressions of aquaporin‐4 and matrix metalloproteinase‐9 were depressed, and expressions of zonula occludens‐1 and occludin were increased significantly, suggesting that CKLF1 may be involved in the destruction of blood–brain barrier after ischemia/reperfusion in cerebral ischemic rats (Kong et al., 2016). The mechanism may be that inhibition of CKLF1 might enhance expressions of tight junction proteins and inhibit expressions of aquaporin‐4 and matrix metalloproteinase‐9, which all maintain the integrity of the blood–brain barrier, thus protecting the neurological function (Kong et al., 2016). Based on the above studies, CKLF1 may be implicated in relevant pathophysiological processes after acute brain injury.

Our study found that serum CKLF1 levels were significantly elevated in patients with sTBI compared to controls, and therefore it is hypothesized that CKLF1 in peripheral blood may partially originate from damaged brain tissue. We investigated the associations of serum CKLF1 levels with trauma severity and prognosis through a linear regression model, a binary logistic regression model, and a Cox proportional hazard model and found that serum CKLF1 levels were independently correlated with GCS scores and Rotterdam CT classification and were independently associated with 180‐day mortality and poor prognosis in sTBI patients. In current clinical studies, GCS scores and Rotterdam CT classification are often used to assess trauma severity and predict the prognosis of patients (Jiang et al., 2022; Yang et al., 2021; Zhang et al., 2023). We therefore hypothesized that serum CKLF1 levels may be strongly associated with trauma severity, 180‐day mortality, and the long‐term function prognosis of sTBI.

There are two limitations in this study. First, this study aimed to demonstrate the prognostic predictive role of serum CKLF1 levels in sTBI, but the sample size was not large enough, and a larger cohort study is needed at a later stage to validate the findings of this study. Second, serum CKLF1 levels were measured by ELISA in this study, and more sensitive LC–MS techniques could be used to measure serum CKLF1 levels in subsequent studies.

## 5 CONCLUSION

In this study, the associations of serum CKLF1 levels with disease severity, 180‐day functional prognosis, and 180‐day mortality in sTBI patients were analyzed for the first time using the multivariate analyses. High serum CKLF1 levels were independently associated with GCS scores, Rotterdam CT classification, and 180‐day poor prognosis. Meanwhile, serum CKLF1 levels had similar prognostic predictive ability, as compared with GCS scores and Rotterdam CT classification. Taken together, serum CKLF1 may be a potential biomarker for assessing severity and predicting long‐term functional outcome and death following sTBI.

## AUTHOR CONTRIBUTIONS

**Xiaoyu Wu**: Conceptualization; investigation; methodology; project administration; writing—original draft. **Chang Su**: Data curation; validation. **Da Tian**: Formal analysis; investigation; supervision. **Yufei Ye**: Data curation; formal analysis; funding acquisition. **Qinghua Du**: Data curation; software. **Junxia Chen**: Resources; validation. **Huguang Li**: Data curation; validation; visualization. **Jin Liu**: Conceptualization; funding acquisition; writing—review and editing.

## CONFLICT OF INTEREST STATEMENT

The authors declare that they have no conflicts of interest.

## FUNDING INFORMATION

Key research and development projects of Zhejiang Provincial, Grant Number 2020C03071; Medical and Health Research Project of Zhejiang Province, 2023XY260; City‐level public welfare technology application research project of Lishui, 2021SJZC086, 2021SJZC080, 2023SJZC078, and 2023SJZC101; Public Welfare Technology Research Program of Lishui, 2022GYX24.

### PEER REVIEW

The peer review history for this paper is available at https://publons.com/publon/10.1002/brb3.3522.

## Supporting information

Table S1 Factors correlated with extended Glasgow outcome scale scores after severe traumatic brain injury.
Table S2 Correlations between extended Glasgow outcome scale scores and other variables using linear regression analysis in severe traumatic brain injury.
Table S3 Factors associated with 180‐day mortality in severe traumatic brain injury.
Table S4 Factors associated with 180‐day function prognosis in severe traumatic brain injury.

Supporting Information

## Data Availability

The data that support the findings of this study are available on request from the corresponding author. The data are not publicly available due to privacy or ethical restrictions.
