# Supplementary material for: Utility of serum chemokine–like factor 1 as a biomarker of severity and prognosis after severe traumatic brain injury: A prospective observational study
Source: Brain Behav. 2024 May 21;14(5):e3522. doi: 10.1002/brb3.3522 (PMC11109498; doi:10.1002/brb3.3522)
Supplement: Supplementary file 1 — Table S1 Factors correlated with extended Glasgow outcome scale scores after severe traumatic brain injury. Table S2 Correlations between extended Glasgow outcome scale scores and other variables using linear regression analysis in severe traumatic brain injury. Table S3 Factors associated with 180‐day mortality in severe traumatic brain injury. Table S4 Factors associated with 180‐day function prognosis in severe traumatic brain injury. [file BRB3-14-e3522-s002.docx]

Supplemental table 1 Factors correlated with extended Glasgow Outcome Scale scores after severe traumatic brain injury

|  | *ρ* | *P* value |
| --- | --- | --- |
| Gender (male/female) | 0.060 | 0.518 |
| Age (y) | -0.026 | 0.780 |
| Current cigarette smoking | -0.071 | 0.444 |
| Alcohol abuse | -0.038 | 0.681 |
| Hypertension | -0.095 | 0.305 |
| Diabetes mellitus | -0.058 | 0.532 |
| Hyperlipidemia | -0.034 | 0.713 |
| Hospital admission time (h) | 0.141 | 0.127 |
| Blood-sampling time (h) | 0.137 | 0.138 |
| Systolic arterial pressure (mmHg) | 0.166 | 0.072 |
| Diastolic arterial pressure (mmHg) | 0.112 | 0.227 |
| Traumatic causes | 0.039 | 0.671 |
| GCS scores | 0.655 | *<0.001 |
| Rotterdam CT classification | -0.680 | *<0.001 |
| Midline shift > 5 mm | -0.227 | *0.013 |
| Abnormal cisterns | -0.228 | *0.012 |
| Epidural hematoma | -0.280 | *0.002 |
| Subdural hematoma | -0.201 | *0.028 |
| Subarachnoid hemorrhage | -0.169 | 0.066 |
| Intraventricular hemorrhage | -0.048 | 0.603 |
| Intracerebral hematoma | -0.104 | 0.259 |
| Brain contusion | -0.173 | 0.060 |
| Pneumocephalus | 0.045 | 0.628 |
| Blood leucocyte count (×10^9^/l) | -0.198 | *0.031 |
| Serum CKLF1 levels (pg/mL) | -0.609 | *<0.001 |

Note: Correlations were analyzed using Spearman test. CT means computerized tomography; GCS, Glasgow coma scale; CKLF1, chemokine-like factor 1.

Supplemental table 2 Correlations between extended Glasgow Outcome Scale scores and other variables using linear regression analysis in severe traumatic brain injury

|  | Univariate linear regression | | Multivariate linear regression | |
| --- | --- | --- | --- | --- |
|  | *t* | *P* value | *t* | *P* value |
| Gender (male/female) | 0.345 | 0.731 | - | - |
| Age (y) | -0.550 | 0.583 | - | - |
| Current cigarette smoking | -0.750 | 0.455 | - | - |
| Alcohol abuse | -0.653 | 0.515 | - | - |
| Hypertension | -1.064 | 0.289 | - | - |
| Diabetes mellitus | -0.605 | 0.546 | - | - |
| Hyperlipidemia | -0.254 | 0.800 | - | - |
| Hospital admission time (h) | 1.568 | 0.120 | - | - |
| Blood-sampling time (h) | 1.719 | 0.088 | - | - |
| Systolic arterial pressure (mmHg) | 1.331 | 0.186 | - | - |
| Diastolic arterial pressure (mmHg) | 0.981 | 0.329 | - | - |
| Traumatic causes | 0.124 | 0.902 | - | - |
| GCS scores | 9.366 | *<0.001 | 2.161 | *0.033 |
| Rotterdam CT classification | -10.493 | *<0.001 | -3.997 | *<0.001 |
| Midline shift > 5 mm | -2.569 | *0.011 | -0.732 | 0.465 |
| Abnormal cisterns | -2.560 | *0.012 | -0.700 | 0.485 |
| Epidural hematoma | -3.189 | *0.002 | -0.739 | 0.462 |
| Subdural hematoma | -2.078 | *0.040 | -0.790 | 0.431 |
| Subarachnoid hemorrhage | -1.828 | 0.070 | - | - |
| Intraventricular hemorrhage | -0.547 | 0.585 | - | - |
| Intracerebral hematoma | -1.233 | 0.220 | - | - |
| Brain contusion | -1.730 | 0.086 | - | - |
| Pneumocephalus | 0.595 | 0.553 | - | - |
| Blood leucocyte count (×10^9^/L) | -2.157 | *0.033 | 0.160 | 0.873 |
| Serum CKLF1 levels (pg/mL) | -8.355 | *<0.001 | -2.733 | *0.007 |

Note: Correlations were done using linear regression analysis in severe traumatic brain injury. CT means computerized tomography; GCS, Glasgow coma scale; CKLF1, chemokine-like factor 1.

Supplemental table 3 Factors associated with 180-day mortality in severe traumatic brain injury

|  | The alive (n=90) | The death (n=29) | *Z/χ^2^/t* | *P* value |
| --- | --- | --- | --- | --- |
| Gender (male/female) | 49/41 | 16/13 | 0.005 | 0.945 |
| Age (y) | 45 (33-56) | 54 (34-63) | -0.982 | 0.326 |
| Current cigarette smoking | 19 (21.1%) | 8 (27.6%) | 0.524 | 0.469 |
| Alcohol abuse | 26 (28.9%) | 11 (37.9%) | 0.837 | 0.360 |
| Hypertension | 13 (14.4%) | 8 (27.5%) | 2.606 | 0.106 |
| Diabetes mellitus | 11 (12.2%) | 6 (20.7%) | 1.284 | 0.257 |
| Hyperlipidemia | 20 (22.2%) | 5 (17.2%) | 0.328 | 0.567 |
| Hospital admission time (h) | 4.3 (3.6-6.0) | 4.0 (2.4-5.2) | -1.511 | 0.131 |
| Blood-sampling time (h) | 5.9 (4.5-7.7) | 4.5 (3.0-6.8) | -1.576 | 0.115 |
| Systolic arterial pressure (mmHg) | 127 (99-141) | 120 (102-123) | -1.535 | 0.125 |
| Diastolic arterial pressure (mmHg) | 74 (65-84) | 73 (62-78) | -0.635 | 0.525 |
| Traumatic causes |  |  | 2.144 | 0.342 |
| Automobile/motorcycle | 44 (48.9%) | 17 (58.6%) |  |  |
| Fall/jump | 38 (42.2%) | 8 (27.6%) |  |  |
| Others | 8 (8.9%) | 4 (13.8%) |  |  |
| GCS scores | 6 (5-7) | 4 (3-5) | -5.546 | *<0.001 |
| Rotterdam CT classification | 4 (4-5) | 6 (5-6) | -5.824 | *<0.001 |
| Midline shift > 5 mm | 45 (50%) | 21 (72.4%) | 4.461 | *0.035 |
| Abnormal cisterns | 63 (70.0%) | 27 (93.1%) | 6.352 | *0.012 |
| Epidural hematoma | 43 (47.8%) | 21 (72.4%) | 5.355 | *0.021 |
| Subdural hematoma | 49 (54.4%) | 20 (69.0%) | 1.898 | 0.168 |
| Subarachnoid hemorrhage | 55 (61.1%) | 22 (75.9%) | 2.090 | 0.148 |
| Intraventricular hemorrhage | 8 (8.9%) | 5 (17.2%) | 1.572 | 0.210 |
| Intracerebral hematoma | 47 (52.2%) | 20 (69.0%) | 2.499 | 0.114 |
| Brain contusion | 53 (58.9%) | 18 (62.1%) | 0.092 | 0.761 |
| Pneumocephalus | 34 (37.8%) | 12 (41.4%) | 0.120 | 0.729 |
| Blood leucocyte count (×10^9^/l) | 9.0 (6.3-10.4) | 10.8 (7.3-12.0) | -2.080 | *0.038 |
| Serum CKLF1 levels (pg/mL) | 49.90 (41.18-61.37) | 65.28 (56.78-84.58) | -5.042 | *<0.001 |

Note: Qualitative variables were presented as counts (percentages) and were compared for intergroup difference using chi-square test or Fisher exact test as appropriate. Quantitative variables were summarized as medians (upper - lower quartiles) or the means ± standard deviations as appropriate. Intergroup comparisons were done using unpaired Student t test or Mann-Whitney U test where appropriate. CT indicates computerized tomography; GCS, Glasgow coma scale; CKLF1, chemokine-like factor 1.

Supplemental table 4 Factors associated with 180-day function prognosis in severe traumatic brain injury

|  | Good prognosis (n=65) | Poor prognosis (n=54) | *Z/χ^2^/t* | *P* value |
| --- | --- | --- | --- | --- |
| Gender (male/female) | 36/29 | 29/25 | 0.034 | 0.855 |
| Age (y) | 46 (33-56) | 47 (33-59) | -0.366 | 0.715 |
| Current cigarette smoking | 13 (20.0%) | 14 (25.9%) | 0.590 | 0.442 |
| Alcohol abuse | 19 (29.2%) | 15 (27.8%) | 0.232 | 0.630 |
| Hypertension | 9 (13.8%) | 12 (22.2%) | 1.424 | 0.233 |
| Diabetes mellitus | 8 (12.7%) | 9 (16.7%) | 0.458 | 0.499 |
| Hyperlipidemia | 14 (21.5%) | 11 (20.4%) | 0.024 | 0.876 |
| Hospital admission time (h) | 4.2 (3.7-5.9) | 4.1 (2.5-5.7) | -1.223 | 0.221 |
| Blood-sampling time (h) | 5.7 (4.7-7.5) | 5.6 (3.5-7.6) | -1.143 | 0.253 |
| Systolic arterial pressure (mmHg) | 127 (102-141) | 120 (93-132) | -1.935 | 0.053 |
| Diastolic arterial pressure (mmHg) | 75 (66-84) | 73 (57-82) | -1.338 | 0.181 |
| Traumatic causes |  |  | 2.144 | 0.342 |
| Automobile/motorcycle | 34 | 27 |  |  |
| Fall/jump | 28 | 18 |  |  |
| Others | 3 | 9 |  |  |
| GCS scores | 6 (5-7) | 4 (3-5) | -6.593 | *<0.001 |
| Rotterdam CT classification | 4 (3-4) | 5 (4-6) | -6.932 | *<0.001 |
| Midline shift > 5 mm | 29 (44.6%) | 37 (68.5%) | 6.823 | *0.009 |
| Abnormal cisterns | 42 (64.6%) | 48 (88.9%) | 9.429 | *0.002 |
| Epidural hematoma | 28 (43.1%) | 36 (66.7%) | 6.603 | *0.010 |
| Subdural hematoma | 33 (50.8%) | 36 (66.7%) | 3.060 | 0.080 |
| Subarachnoid hemorrhage | 37 (56.9%) | 40 (74.1%) | 3.799 | 0.051 |
| Intraventricular hemorrhage | 6 (9.2%) | 7 (13.0%) | 0.422 | 0.516 |
| Intracerebral hematoma | 33 (50.8%) | 34 (63.0%) | 1.783 | 0.182 |
| Brain contusion | 34 (52.3%) | 37 (68.5%) | 3.221 | 0.073 |
| Pneumocephalus | 27 (41.5%) | 19 (35.2%) | 0.502 | 0.479 |
| Blood leucocyte count (×10^9^/L) | 8.1 (6.2-10.2) | 10.2 (1.0-12.0) | -2.376 | 0.018 |
| Serum CKLF1 levels (pg/mL) | 47.60 (34.68-58.99) | 62.73 (54.78-75.48) | -5.818 | *<0.001 |

Note: Qualitative variables were presented as counts (percentages) and were compared for intergroup difference using chi-square test or Fisher exact test as appropriate. Quantitative variables were summarized as medians (upper - lower quartiles) or the means ± standard deviations as appropriate. Intergroup comparisons were done using unpaired Student t test or Mann-Whitney U test where appropriate. CT indicates computerized tomography; GCS, Glasgow coma scale; CKLF1, chemokine-like factor 1.
