# Supplementary material for: Utility of serum chemokine–like factor 1 as a biomarker of severity and prognosis after severe traumatic brain injury: A prospective observational study
Source: Brain Behav. 2024 May 21;14(5):e3522. doi: 10.1002/brb3.3522 (PMC11109498; doi:10.1002/brb3.3522)
Supplement: Supplementary file 2 — Supporting Information [file BRB3-14-e3522-s001.docx]

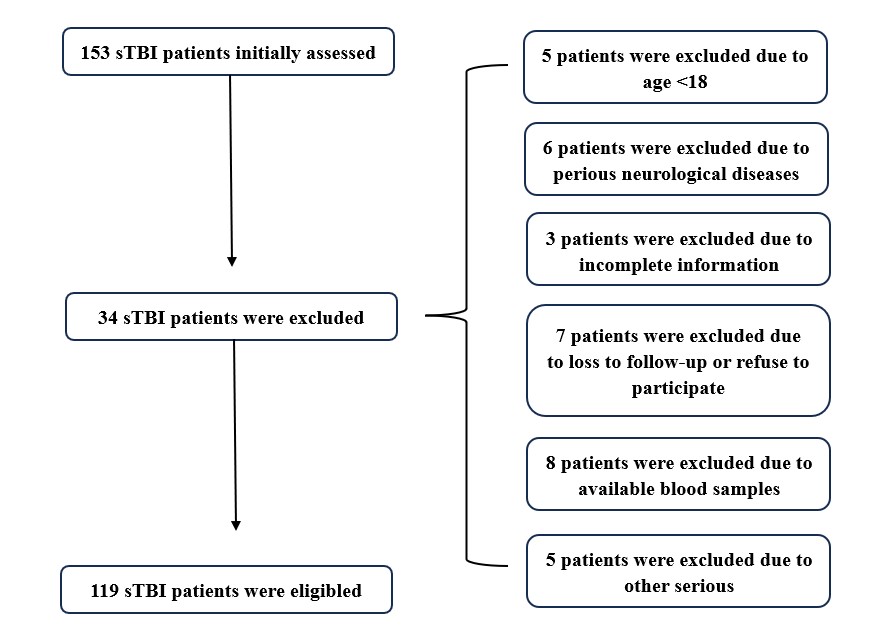


**Supplemental Figure 1**


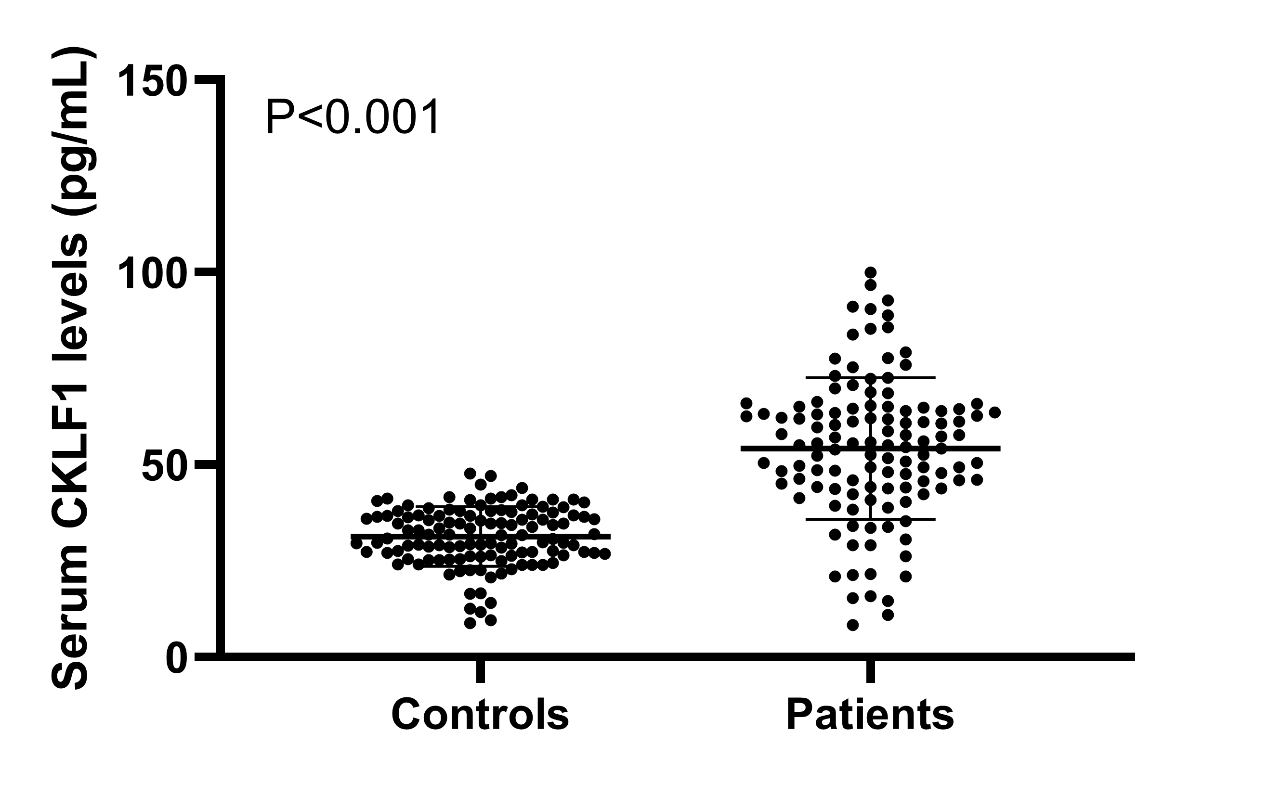


**Supplemental Figure 2**


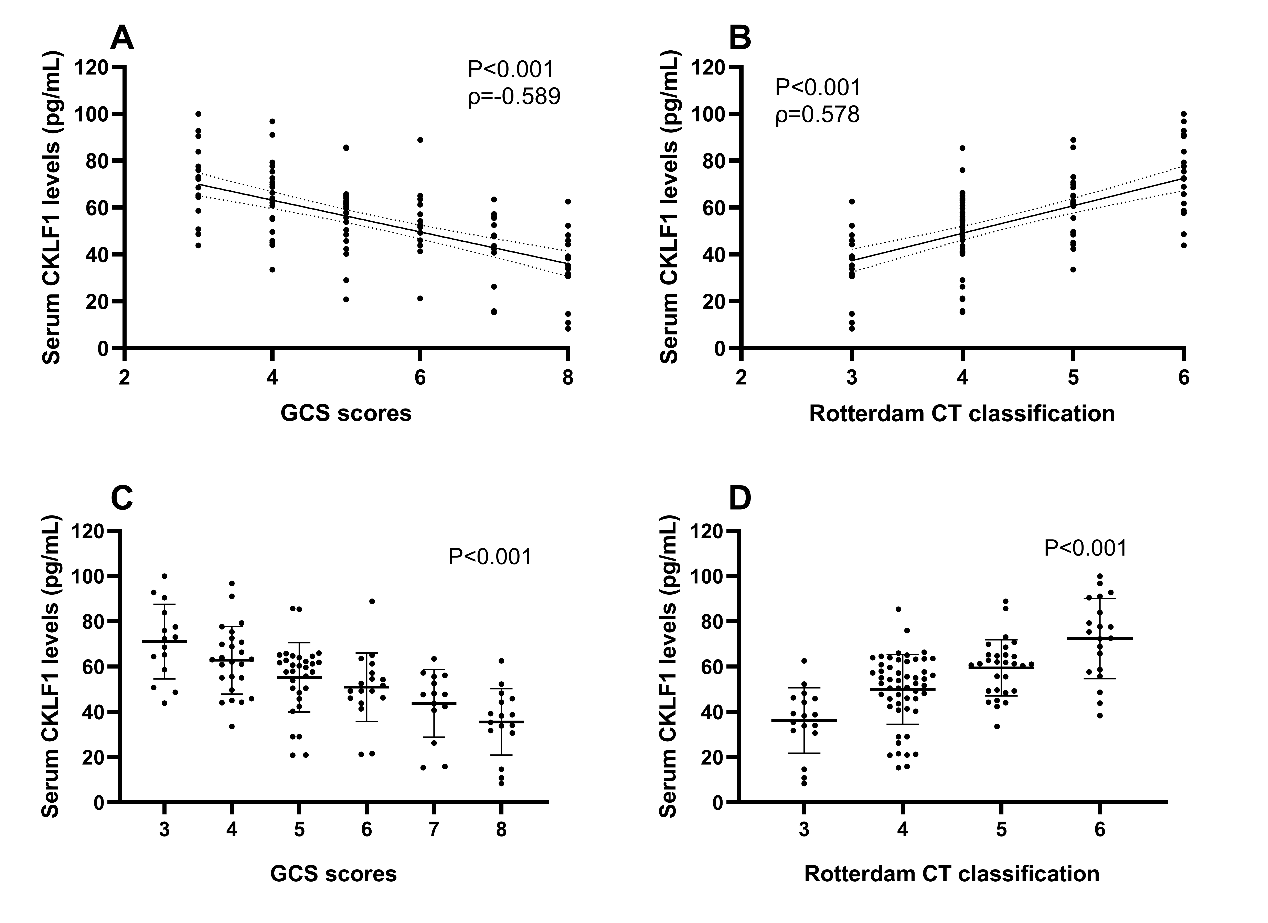


**Supplemental Figure 3**


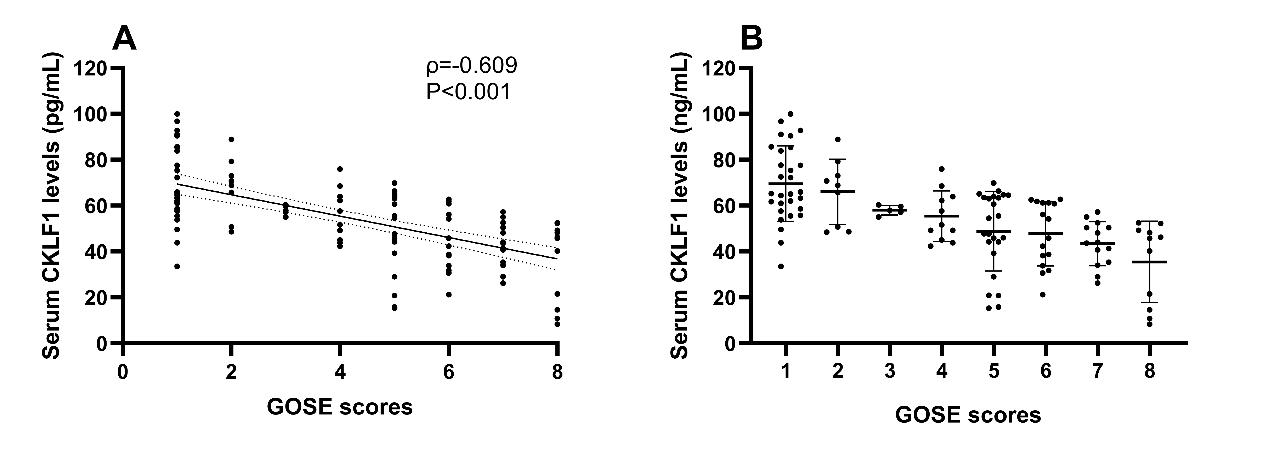


**Supplemental Figure 4**


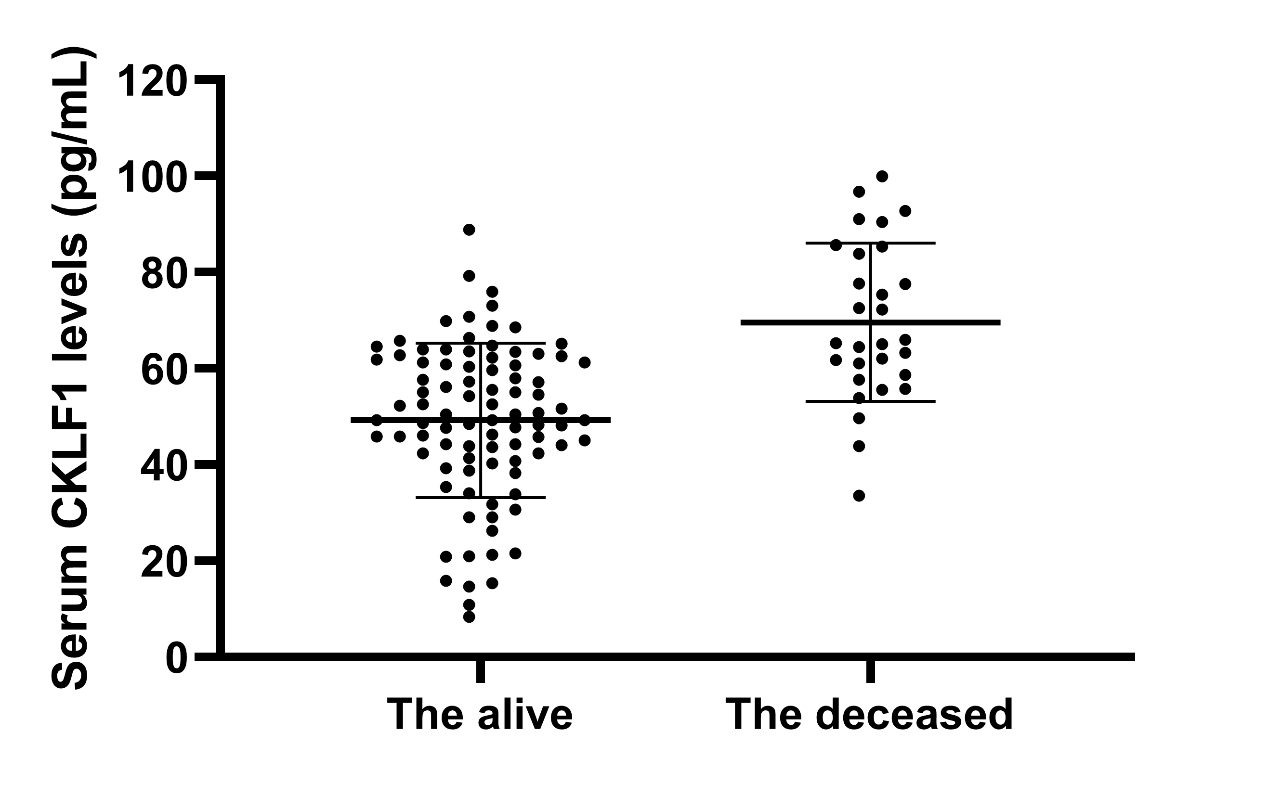


**Supplemental Figure 5**


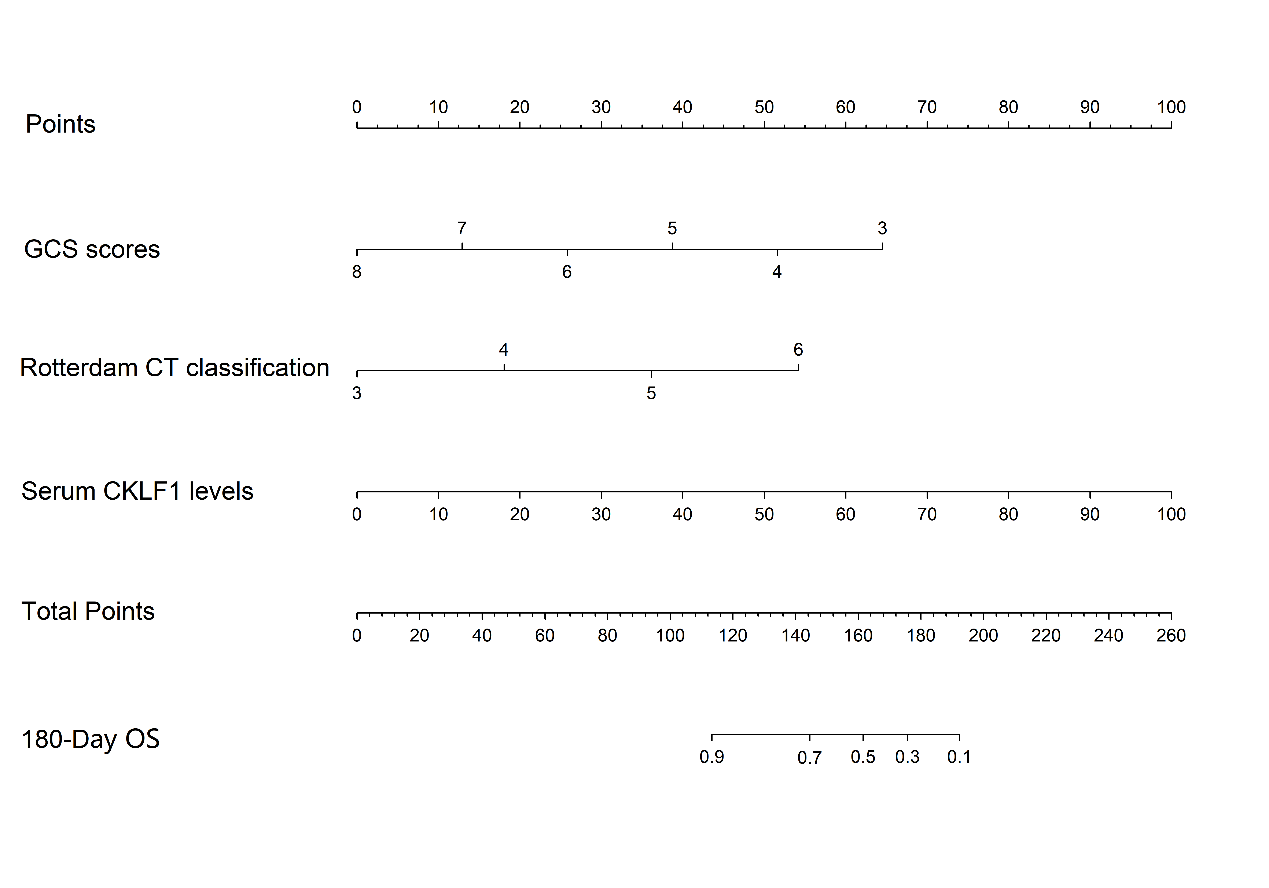


**Supplemental Figure 6**


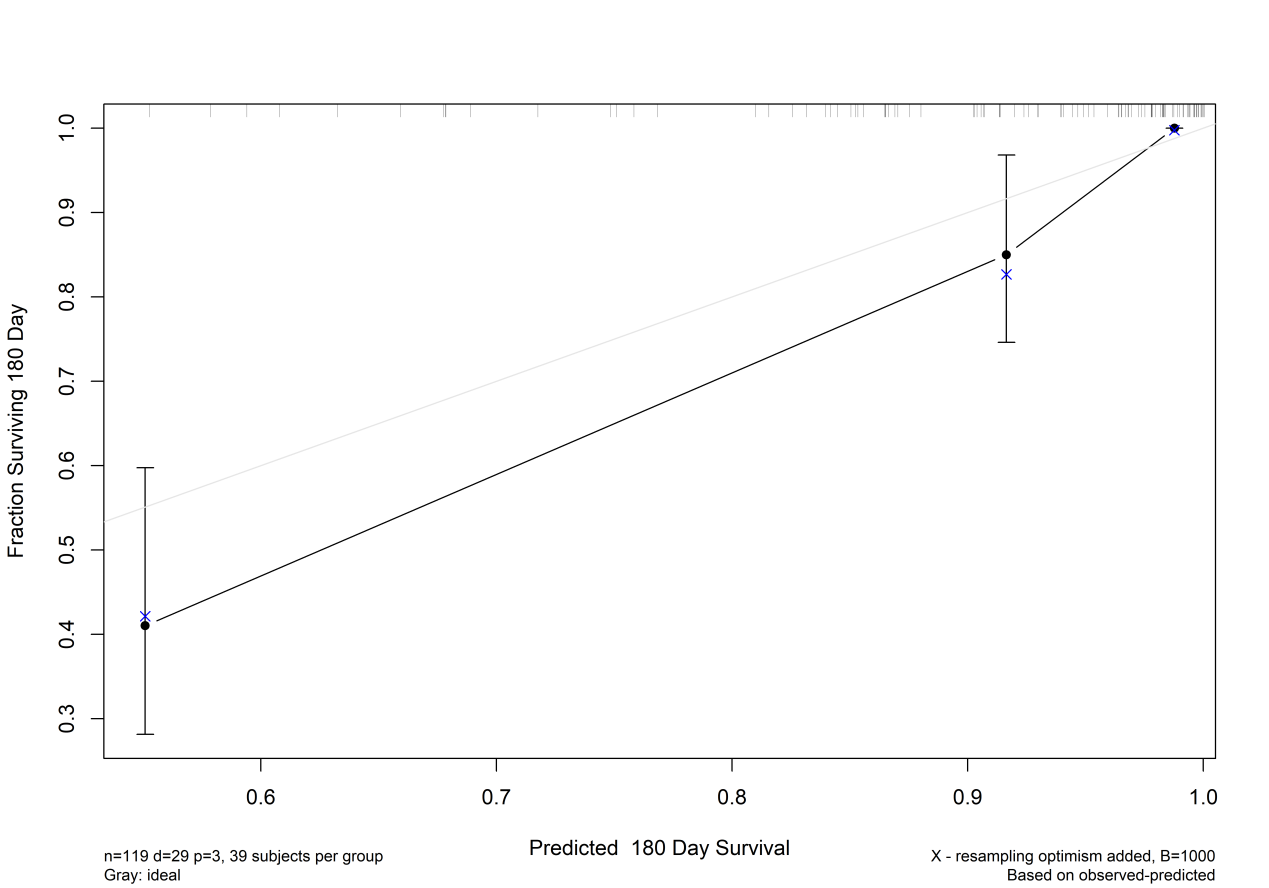


**Supplemental Figure 7**


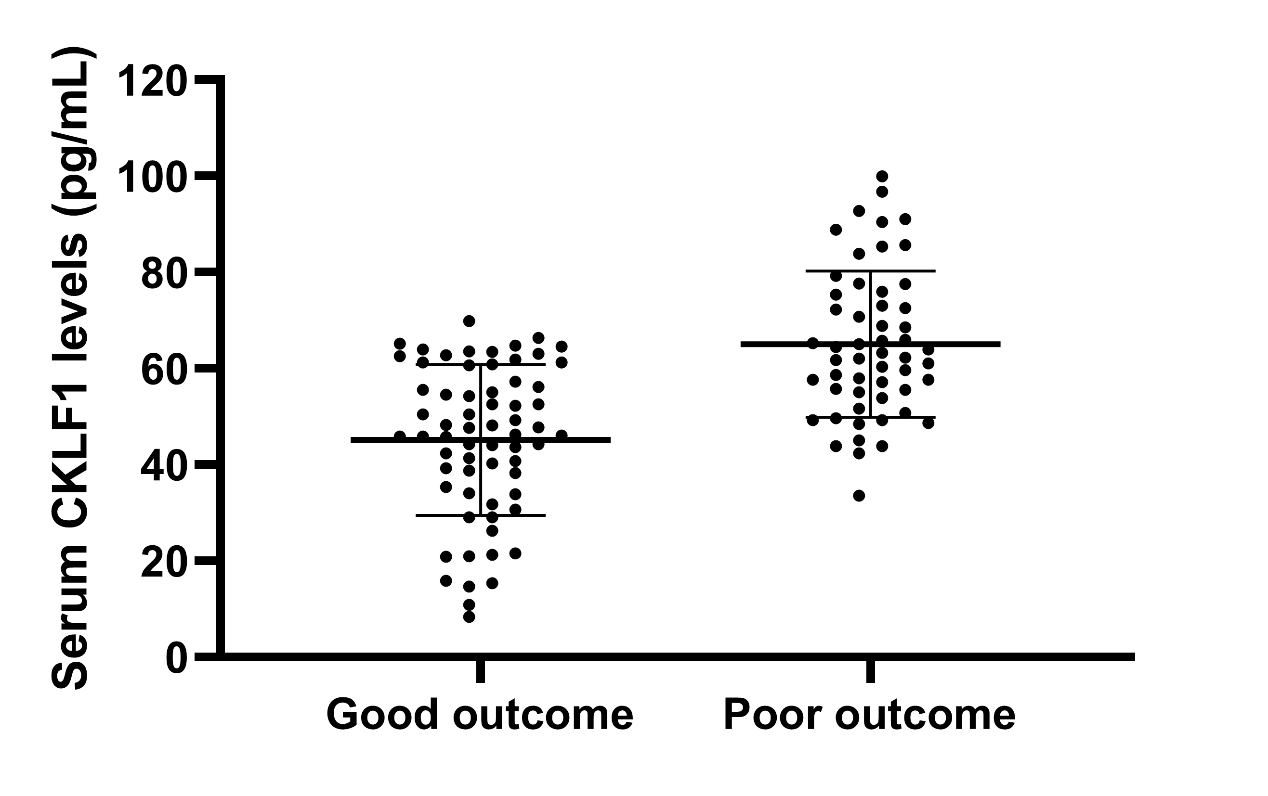


**Supplemental Figure 8**


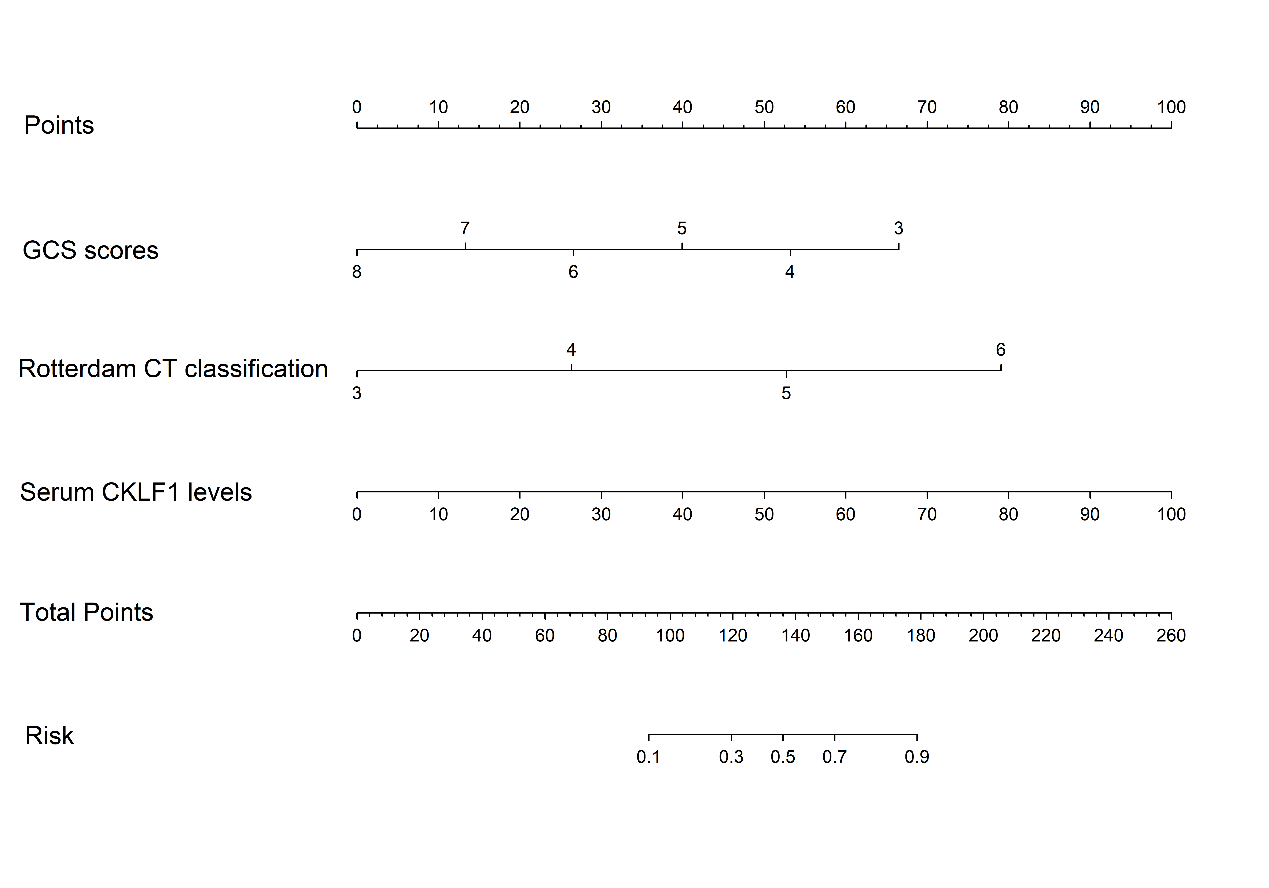


**Supplemental Figure 9**


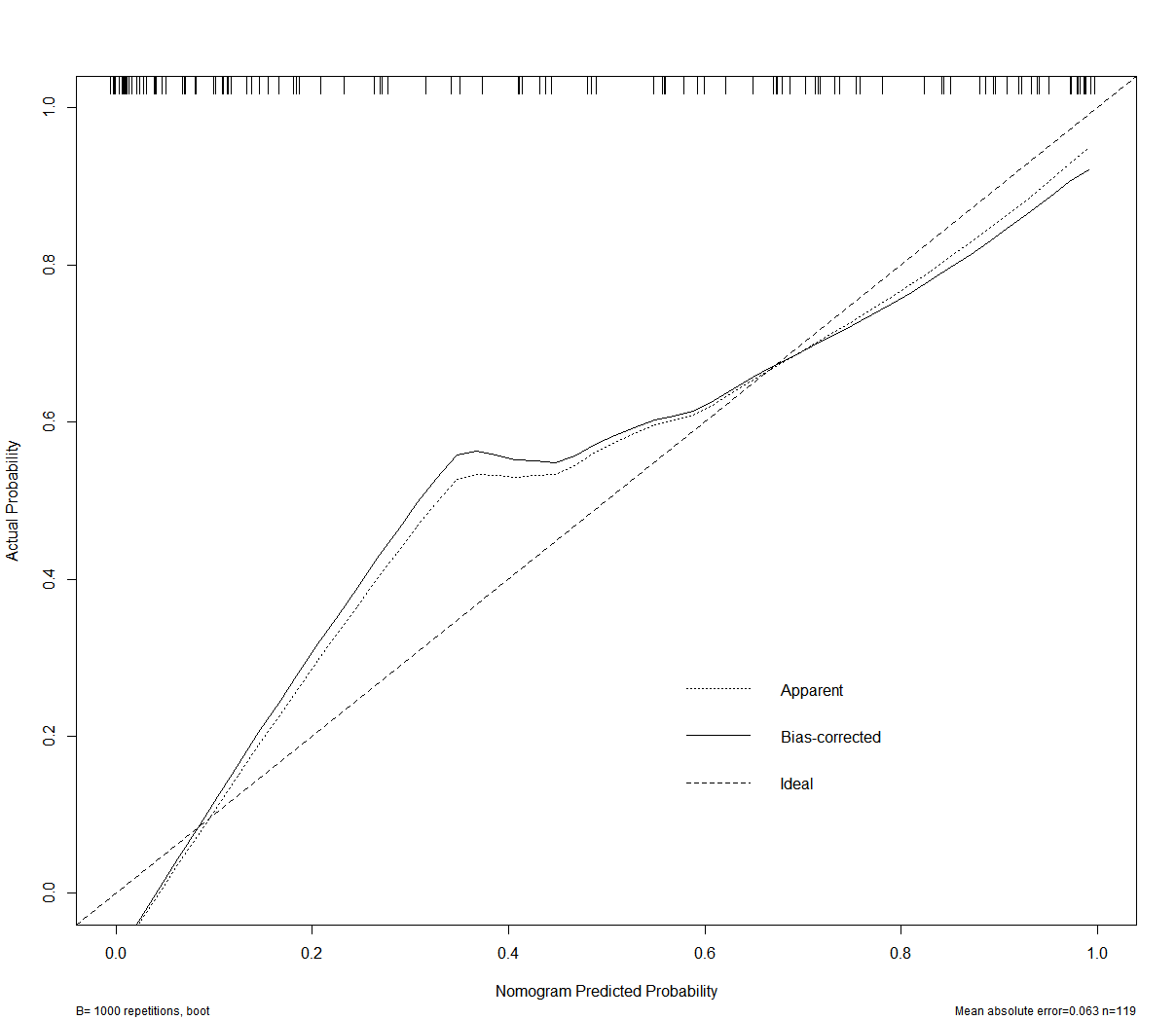


**Supplemental Figure 10**

**Supplemental Figure 1**

Flowing-chart for selecting eligible patients with severe traumatic brain injury.

Initially, a total of 153 patients were assessed and ultimately, 119 patients were analyzed after excluding 34 patients in compliance with the exclusion criteria.

sTBI denotes severe traumatic brain injury.

**Supplemental Figure 2**

Boxplot illustrating admission serum chemokine-like factor 1 levels between patients with severe traumatic brain injury and controls.

Data were reported as median (upper-lower quartiles) and two groups were compared using the Mann-Whitney U-test. Serum chemokine-like factor 1 levels at admission were significantly higher in patients with severe traumatic brain injury than in controls (P<0.001)

CKLF1 denotes chemokine-like factor 1.

**Supplemental Figure 3**

Boxplots and correlograms describing relationships between admission serum chemokine-like factor 1 levels and traumatic severity of severe traumatic brain injury.

A: Correlograms illustrating the relationship between admission serum chemokine-like factor 1 levels and baseline Glasgow coma scale scores after severe traumatic brain injury.

Using the Spearman correlation coefficient, admission serum chemokine-like factor 1 levels were tightly negatively correlated with baseline Glasgow coma scale scores after severe traumatic brain injury (P<0.001).

B: Correlograms illustrating the relationship between admission serum chemokine-like factor 1 levels and baseline Rotterdam computerized tomography classification after severe traumatic brain injury.

Using the Spearman correlation coefficient, admission serum chemokine-like factor 1 levels were highly positively correlated with baseline Rotterdam computerized tomography classification after severe traumatic brain injury (P<0.001).

C: Boxplots illustrating admission serum chemokine-like factor 1 levels among subgroups based on baseline Glasgow coma scale scores after severe traumatic brain injury.

Patients were divided into six groups in accordance with baseline Glasgow coma scale scores, namely, scores 3 (n = 15), 4 (n = 25), 5 (n = 31), 6 (n = 18), 7 (n = 14) and 8 (n = 16). Using the Kruskal-Wallis H-test, admission serum chemokine-like factor 1 levels were significantly declined in the order of baseline Glasgow coma scale scores from 3 to 8 (P<0.001).

D: Boxplots illustrating admission serum chemokine-like factor 1 levels among subgroups based on baseline Rotterdam computerized tomography classification after severe traumatic brain injury.

Patients were divided into four groups in accordance with baseline Rotterdam computerized tomography classification, namely, scores 3 (n = 17), 4 (n = 53), 5 (n = 29) and 6 (n = 20). Using the Kruskal-Wallis H-test, admission serum chemokine-like factor 1 levels were significantly raised in the order of baseline Rotterdam computerized tomography classification from 3 to 6 (P<0.001).

CKLF1 denotes chemokine-like factor 1; GCS, Glasgow coma scale; CT, computerized tomography.

**Supplemental Figure 4**

Boxplot and correlogram describing relationships between admission serum chemokine-like factor 1 levels and 180-day prognosis of severe traumatic brain injury.

A: Correlogram illustrating the relationship between admission serum chemokine-like factor 1 levels and 180-day extended Glasgow Outcome Scale scores after severe traumatic brain injury.

Using the Spearman correlation coefficient, admission serum chemokine-like factor 1 levels were markedly inversely correlated with 180-day extended Glasgow Outcome Scale scores after severe traumatic brain injury (P<0.001).

B: Boxplot illustrating admission serum chemokine-like factor 1 levels among patients with different extended Glasgow Outcome Scale scores at 180 days after severe traumatic brain injury.

Patients were assigned into eight groups in accordance with different extended Glasgow Outcome Scale scores at 180 days after severe traumatic brain injury. Using the Kruskal-Wallis H-test, admission serum chemokine-like factor 1 levels were substantially decreased in the order of extended Glasgow Outcome Scale scores at 180 days after severe traumatic brain injury (P<0.001).

CKLF1 denotes chemokine-like factor 1; GOSE, Extended Glasgow Outcome Scale.

**Supplemental Figure 5**

Boxplot illustrating baseline serum chemokine-like factor 1 levels between the deceased and the alive at 180 days after severe traumatic brain injury.

Data were reported as median (upper-lower quartiles). Using the Mann-Whitney U-test, the deceased patients had significantly higher baseline serum chemokine-like factor 1 levels than the alive ones (P<0.001).

CKLF1 denotes chemokine-like factor 1.

**Supplemental Figure 6**

Nomogram assessing risk of 180-day overall survival after serve traumatic brain injury.

The overall survival prediction combined model, in which admission serum chemokine-like factor 1 levels, baseline Glasgow coma scale scores and baseline Rotterdam computerized tomography classification were incorporated, was displayed via a nomogram.

CKLF1 denotes chemokine-like factor 1; GCS, Glasgow coma scale; CT, computerized tomography; OS, overall survival.

**Supplemental Figure 7**

Calibration curve assessing reliability of 180-day overall survival prediction model after serve traumatic brain injury.

Under calibration curve, the combined model incorporating admission serum chemokine-like factor 1 levels, baseline Glasgow coma scale scores and baseline Rotterdam computerized tomography classification, was comparatively steady.

**Supplemental Figure 8**

Boxplot illustrating admission serum chemokine-like factor 1 levels between patients with good prognosis and those with poor prognosis at 180 days after severe traumatic brain injury.

Poor prognosis was defined as extended Glasgow Outcome Scale scores of 1--4 at 180 days after severe traumatic brain injury. Data were reported as median (upper-lower quartiles). Using the Mann-Whitney U-test, admission serum chemokine-like factor 1 levels were substantially higher in patients with poor prognosis than in those with good prognosis at 180 days after severe traumatic brain injury.

CKLF1 denotes chemokine-like factor 1.

**Supplemental Figure 9**

Nomogram predicting poor prognosis after serve traumatic brain injury.

The poor prognosis prediction combined model, in which admission serum chemokine-like factor 1 levels, baseline Glasgow coma scale scores and baseline Rotterdam computerized tomography classification were incorporated, was displayed via a nomogram.

CKLF1 denotes chemokine-like factor 1; GCS, Glasgow coma scale; CT, computerized tomography.

**Supplemental Figure 10**

Calibration curve assessing reliability of prognosis prediction model after server traumatic brain injury.

Under calibration curve, the prognosis prediction combined model containing admission serum chemokine-like factor 1 levels, baseline Glasgow coma scale scores and baseline Rotterdam computerized tomography classification, was relatively stable.
